# Immunotherapeutics Combining a Recombinant Chimeric Protein, Monophosphoryl Lipid A, and Miltefosine Against Visceral Leishmaniasis

**DOI:** 10.3390/pathogens14121202

**Published:** 2025-11-25

**Authors:** Marcelo M. Jesus, Daniela P. Lage, Breno L. Pimenta, Gabriel J. L. Moreira, Isabela A. G. Pereira, Raquel S. B. Câmara, Ana L. Silva, Grasiele S. V. Tavares, Karolina O. M. Falcão, Saulo S. G. Dias, Dóris M. Abrão, Maíza M. Rodrigues, João A. Oliveira-da-Silva, Mário S. Giusta, Miguel A. Chávez-Fumagalli, Bruno M. Roatt, Alexsandro S. Galdino, Myron Christodoulides, Camila S. Freitas, Eduardo A. F. Coelho

**Affiliations:** 1 Laboratório de Pesquisa do Programa de Pós-Graduação em Ciências da Saúde, Infectologia e Medicina Tropical, Faculdade de Medicina, Universidade Federal de Minas Gerais, Avenida Alfredo Balena, 190, Belo Horizonte 30130-100, Minas Gerais, Brazil; marcelobiot@gmail.com (M.M.J.); amorim.gpereira@gmail.com (I.A.G.P.);; 2 Laboratório de Imunopatologia, Núcleo de Pesquisas em Ciências Biológicas/NUPEB, Departamento de Ciências Biológicas, Insituto de Ciências Exatas e Biológicas, Universidade Federal de Ouro Preto, Ouro Preto 35402-136, Minas Gerais, Brazilroatt@ufop.edu.br (B.M.R.); 3 Departamento de Patologia Clínica, COLTEC, Universidade Federal de Minas Gerais, Av. Antônio Carlos, 6627, Belo Horizonte 31270-901, Minas Gerais, Brazil; 4 Computational Biology and Chemistry Research Group, Vicerrectorado de Investigación, Universidad Católica de Santa María, Urb. San José S/N, Umacollo, Arequipa 04000, Peru; mchavezf@ucsm.edu.pe; 5 Laboratório de Biotecnologia de Microrganismos, Universidade Federal de São João Del-Rei, Divinópolis 35501-296, Minas Gerais, Brazil; 6 Neisseria Research Group, Molecular Microbiology, School of Clinical and Experimental Sciences, Faculty of Medicine, University of Southampton, Southampton General Hospital, Southampton SO16 6YD, UK; m.christodoulides@soton.ac.uk

**Keywords:** immunochemotherapy, visceral leishmaniasis, chimeric protein, monophosphoryl lipid-A, miltefosine, immune response

## Abstract

Current treatment for visceral leishmaniasis (VL) is associated with toxicity, a high cost, and the emergence of drug-resistant parasite strains. Moreover, no human vaccine is available. In this context, immunotherapeutics combining vaccination and chemotherapy have emerged as a promising alternative. In this study, we combined a recombinant chimeric protein (ChimT) with the adjuvant 3-*O*-desacyl–monophosphoryl lipid A (MPLA) and the antileishmanial drug miltefosine (Milt) and used it to treat *Leishmania infantum*-infected mice. Parasitological, immunological, and toxicological assays were performed at 1 and 30 days post treatment. The ChimT/MPLA/Milt combination was the most effective therapeutic regimen, inducing robust Th1-type cellular and humoral immune responses, as demonstrated by increased levels of IFN-γ, IL-12, and TNF-α cytokines, nitrite, and IgG2a antibodies. These responses were associated with significant reductions in parasite load across various organs. Furthermore, the treatment showed low renal and hepatic toxicity at both evaluation time points. By contrast, ChimT alone, ChimT/MPLA, and Milt induced lower immunological and parasitological responses when compared with the ChimT/MPLA/Milt group. The results observed at 1 and 30 days post treatment demonstrated the potential of ChimT/MPLA/Milt combination against VL.

## 1. Introduction

Leishmaniasis is a tropical disease caused by infection with *Leishmania* parasites, which is endemic in 98 countries in the world, with 380 million people exposed to the risks of infection [1]. The main clinical manifestations are defined as tegumentary (TL) and visceral (VL) leishmaniasis. TL includes the cutaneous (CL), cutaneous-diffuse (DCL), and mucosal (ML) forms, which cause self-limiting lesions that can progress to tissue and/or mucosal destruction and patient morbidity [2]. VL represents the second most challenging infectious disease worldwide, with 500,000 new cases and 60,000 deaths annually [3]. Treatment against VL uses pentavalent antimonials, pentamidine, amphotericin B (AmpB), paromomycin, and miltefosine, amongst others [4,5]. However, they cause organic toxicity, are expensive, and their use is blighted by the emergence of drug-resistant strains [6]. Miltefosine was developed as an anticancer agent, and its antileishmanial activity was demonstrated more recently, being the only drug that can be administered orally [7,8,9,10]. It disrupts lipid metabolism and affects cell signaling pathways and parasite membrane synthesis. Miltefosine causes parasite death due to the interaction with the acidocalcisome and the stimulation of the sphingosine-dependent plasma membrane calcium channels [11]. Although effective, miltefosine is not recommended for pregnant women [12] and resistant strains have been identified in India and Brazil [13]. In this context, as the repertoire of non-toxic and effective drugs is limited, there is a need to find alternate options for treating VL.

Vaccination is one cost-effective option that could be used as a control measure against leishmaniasis [14]. Although candidates have been evaluated in mice [15,16], hamsters [17,18], and dogs [19,20], most of them are still in the early stages of development. Consequently, there is no human vaccine against VL. A strategy combining vaccination and treatment, termed immunochemotherapy or immunotherapy, has emerged as an alternative to reduce the parasitism and activate the host immune system [21,22,23]. Considering the genetic diversity of *Leishmania* parasites and the polymorphic nature of the mammalian hosts, the development of polypeptide-based vaccines can be favored, since they present distinct T cell epitopes from parasite proteins, which improve immunogenicity and protective efficacy over the use of single proteins [24,25]. Immunoproteomics approaches have been used to identify antigenic and immunogenic *Leishmania* proteins [26,27].

Studies have revealed immunological candidates including prohibitin (PHB), eukaryotic initiation factor 5a (eIF5a), and the hypothetical LiHyp1 and LiHyp2 proteins [28,29]. In fact, mice immunized with PHB vaccine developed a Th1-type immune response characterized by the production of IFN-γ, IL-12, and GM-CSF cytokines, in addition to nitrite and IgG2a antibodies [30]. Immunized mice showed a low burden of parasites in organs when compared to unvaccinated control mice. The amino acid sequence from the recombinant eIF5a protein contained T cell epitopes that were used to construct a chimeric protein, which induced partial protection in immunized mice against *L. infantum* infection. Such protection was also based on the production of Th1-type cytokines by both CD4^+^ and CD8^+^ T cells [27]. In another immunoproteomics study, the *Leishmania* LiHyp1 and LiHyp2 proteins were recognized by antibodies in sera from VL patients, suggesting immunological potential [29].

Previously, we applied bioinformatics tools to predict and select T cell epitopes from the amino acid sequences of the PHB, eIF5a, LiHyp1, and LiHyp2 proteins and construct a novel recombinant chimeric protein, ChimT. BALB/c mice were immunized with ChimT and a Th1-type adjuvant and challenged subsequently with *L. infantum* promastigotes. This experimental vaccine induced a positive parasitological and immunological response against infection [27]. Building on these findings, ChimT was evaluated in combination with the adjuvant 3-*O*-desacyl–monophosphoryl lipid A (MPLA), and a protective immune response against *L. infantum* infection was also found in vaccinated mice [31]. As an example of immunotherapy, a combination of ChimT, MPLA, and AmpB was used to treat *L. infantum*-infected mice [27]. This immunotherapy induced the development of Th1-biased cellular and humoral responses, with high levels of IFN-γ and IL-12, elevated IFN-γ mRNA expression, and increased IgG2a:IgG1 production, which all contributed to reducing the parasite burden in animal organs. As a consequence, in the current study, we aimed to refine the immunotherapy method combining ChimT with MPLA, but replacing AmpB with miltefosine (Milt) as the antileishmanial drug to treat *L. infantum*-infected mice. The rationale for this was to introduce AmpB of known toxicity for mammals [32,33,34]. Thus, we tested the hypothesis that a new combination immunotherapy for VL consisting of ChimT/MPLA/Milt would induce a Th1-type immune response in mice, which leads to reduced parasitism accompanied by low organic toxicity.

## 2. Materials and Methods

### 2.1. Mice, Parasites, and Experimental Infection

This study was approved by the Federal University of Minas Gerais (UFMG, Belo Horizonte, Brazil) Committee on Ethical Handling of Research Animals (protocol 144/2020). Female BALB/c mice (8 weeks old) were obtained from the Bioterism Center of UFMG, and were maintained under specific pathogen-free conditions, with food and water provided ad libitum. *Leishmania infantum* (MHOM/BR/1974/PP75) stationary promastigotes were cultured according to [35]. Soluble *Leishmania* antigen extract (SLA) was prepared with 1 × 10^9^ stationary promastigotes, which were washed three times with phosphate-buffered saline (PBS 1x) and subjected to six cycles of freezing in liquid nitrogen and thawing in water bath, followed by ultrasonication (Ultrasonic Processor GEX600) for five cycles of 30 s at 38 MHz. After centrifugation at 9000× *g* for 30 min at 4 °C, the supernatant containing SLA was collected and stored at −70 °C. Protein concentration was determined using the BCA Protein Assay Kit (Thermo Scientific, Waltham, MA, USA), according to the manufacturer’s instructions. For infection, mice were inoculated subcutaneously with 1 × 10^6^ stationary *L. infantum* promastigotes [36].

### 2.2. Expression and Purification of the ChimT Protein

Recombinant chimeric protein ChimT was produced as previously described [35]. Protein expression was induced with 0.5 mM isopropyl β-D-1-thiogalactopyranoside (IPTG; Sigma-Aldrich, Burlington, MA, USA), and cultures were incubated at 12 °C for 24 h with agitation (200× *g*). Cells were lysed by ultrasonication (six cycles of 30 s at 38 MHz), followed by six freeze–thaw cycles, and cellular debris was removed by centrifugation. The ChimT protein was purified in a HisTrap HP affinity column (GE Healthcare, Chicago, IL, USA) connected to an ÄKTA system. Eluted fractions were concentrated using Amicon^®^ Ultra-15 centrifugal filters (10,000 NMWL; Millipore, Darmstadt, Germany) and further purified by gel filtration chromatography using a Superdex™ 200 column (GE Healthcare Life Sciences, Marlborough, MA, USA). Endotoxin was removed on a polymyxin B-agarose column, and residual endotoxin levels were quantified using the Pierce™ Chromogenic Endotoxin Quant Kit (Thermo Scientific, Waltham, MA, USA). Final endotoxin levels were <10 ng of lipopolysaccharide per mg of protein. To evaluate the purity of ChimT, total bacterial extracts containing the expressed protein and the protein obtained at the end of the purification process (10 µg each sample) were examined with 12% SDS-PAGE, with Coomassie blue staining (Supplementary Figure S1).

### 2.3. Experimental Groups and Treatment Protocols

Miltefosine (Milteforan™) was purchased from Virbac^®^, Carros Cedex, France, and the formulation was an aqueous oral solution containing 2% drug dissolved in water and propylene glycol, with benzoate preservatives and flavoring agents. No additional solvent was required for administration and MPLA was purchased from Sigma-Aldrich, Burlington, MA, USA. Two experiments were performed and data shown here are representative of the first experiment. In each experiment, eight animals per group were used in the analyses. In this context, \BALB/c mice were divided randomly into seven experimental groups, as follows: (1) Naive (uninfected and untreated) mice: these mice received sterile PBS by oral gavage for 28 consecutive days. (2) Saline (infected and untreated) mice: these mice were infected and received sterile PBS by oral gavage for 28 consecutive days. (3) MPLA mice: these mice were infected and treated subcutaneously with MPLA (20 μg) in four doses, administered at 7-day intervals. (4) ChimT mice: these mice were infected and treated subcutaneously with ChimT (20 µg) in four doses, administered at 7-day intervals. (5) Miltefosine (Milt) mice: these mice were infected and treated orally with miltefosine (2% (40 μg) given once daily for six consecutive days). (6) ChimT/MPLA mice: these mice were infected and treated subcutaneously with ChimT (20 µg) and MPLA (20 µg) in four doses, administered at 7-day intervals. (7) ChimT/MPLA/Milt mice: these mice were infected and treated subcutaneously with ChimT (20 µg) and MPLA (20 μg) in four doses, administered at 7-day intervals, and with oral miltefosine (2% (40 μg) for six consecutive days). On day 28, all treatments were completed. On day 29, half of the animals from each group were euthanized for immediate immunological and parasitological analyses. Blood was collected for humoral response assessment, and the spleen was removed for splenocyte culture and the determination of cellular immunity and parasite burden. The livers, draining lymph nodes (dLNs), and bone marrow (BM) were also collected for parasitological evaluation. Thirty days after the end of treatment (day sixty), the remaining animals from each group were euthanized, and the same samples and analyses were performed to assess the long-term immunological and parasitological responses. A summary of the experimental groups and treatment protocols is also shown (Supplementary Figure S2).

### 2.4. Spleen Cell Culture

Splenocytes were isolated from mice as follows: After euthanasia, spleens were aseptically removed, carefully cleaned of surrounding fat, placed in sterile 15 mL conical tubes containing 1 mL of incomplete RPMI 1640 medium (Sigma-Aldrich, Burlington, MA, USA), and kept on ice. Each spleen was homogenized gently, and the suspension was brought to a final volume of 10 mL with incomplete RPMI. The homogenate was centrifuged at 1200× *g* for 10 min at 4 °C, and the pellet was suspended in 3 mL of red blood cell lysis buffer for 4 min. The reaction was stopped by adding 10 mL of incomplete RPMI, followed by another centrifugation at 1200× *g* for 10 min at 4 °C. When necessary, an additional low-speed centrifugation at 500× *g* for 1 min in 4 °C was performed to remove residual splenic capsule debris. The pellet was suspended in 1 mL of complete RPMI 1640 medium supplemented with 10% (*v*/*v*) heat-inactivated FBS and 1% (*v*/*v*) penicillin–streptomycin solution, and viable cells were counted using a Neubauer chamber. The cell concentration was adjusted to the culture stimulation (in 5 × 10^6^ cells/well).

### 2.5. Cytokine Production and Nitrite Secretion

BALB/c mice were euthanized at 1 and 30 days post treatment, and spleens were collected for splenocyte culture. Cultures (5 × 10^6^ cells per well) were plated in duplicate in 24-well plates (Nunc) containing complete RPMI 1640 medium. Cells were either left unstimulated (medium only) or stimulated with ChimT (10 µg/well) or SLA (25 µg/well) for 48 h at 37 °C in a humidified atmosphere with 5% (*v*/*v*) CO_2_. Supernatants were collected, and concentrations of IFN-γ, IL-4, IL-10, and IL-12 were quantified using commercial ELISA kits (OptEIA™ Mouse ELISA Sets; Pharmingen, San Diego, CA, USA; catalog numbers 555138, 555232, 555252, and 555256, respectively) [35]. Supernatants were collected and analyzed for IFN-γ levels. Nitrite production in the same culture supernatant was evaluated using the Griess reaction. Briefly, 50 µL of supernatant was mixed with 50 µL of Griess reagent, and absorbance was measured at 540 nm. Nitrite concentrations were calculated using a standard sodium nitrite curve [37]. To assess CD4^+^ and CD8^+^ T cell contributions to IFN-γ production, splenocytes (5 × 10^6^ cells/well) were stimulated with ChimT (10 µg/well) or SLA (25 µg/well), and incubated in the presence of either anti-CD4 (clone GK 1.5) or anti-CD8 (clone 53-6.7) monoclonal antibody (5 µg/well), for 48 h at 37 °C with 5% (*v*/*v*) CO_2_. Isotype-matched control antibodies (rat IgG2a, clone R35-95; and rat IgG2b, clone 95-1) were included. All antibodies (No Azide/Low Endotoxin™) were purchased from BD Pharmingen (San Diego, CA, USA).

### 2.6. IFN-γ mRNA Expression in Stimulated Cell Cultures

Quantification of IFN-γ mRNA expression was achieved via RT-qPCR, as previously described [38]. The total RNA content was extracted from splenocytes stimulated with SLA using TRIzol™ reagent (Invitrogen, Waltham, MA, USA). RNA was suspended in UltraPure™ DNase/RNase-Free Distilled Water (Invitrogen, Waltham, MA, USA) and concentration and purity were determined using a NanoDrop Lite spectrophotometer (Thermo Fisher Scientific, Waltham, MA, USA) based on the 260/280 nm absorbance ratio. RNA integrity was confirmed by electrophoresis on a 1.5% agarose gel. Samples were treated with DNase (Invitrogen, Waltham, MA, USA) for 15 min at RT to remove genomic DNA, followed by enzyme inactivation with 25 mM EDTA at 65 °C for 10 min. Complementary DNA (cDNA) was synthesized from 2 µg of RNA using the High-Capacity cDNA Reverse Transcription Kit (Applied Biosystems, Waltham, MA, USA) under the following thermal profile: 25 °C for 10 min, 37 °C for 120 min, and 85 °C for 5 min. RT-qPCR was performed using the PowerUp™ SYBR™ Green Master Mix (Thermo Fisher, Waltham, MA, USA) with gene-specific primers for IFN-γ: forward: 5′-TCAAGTGGCATAGATGTGGAAGAA-3′; reverse: 5′-TGGCTCTGCAGGATTTTCATG-3′. A 7900HT thermocycler (Applied Biosystems, Waltham, MA, USA) was used for amplification with ACTB and GAPDH as housekeeping genes. Primer concentrations (5, 10, and 15 pmol) were optimized for specificity and efficiency. Cycling conditions included an initial denaturation at 95 °C for 10 min, followed by 40 cycles of 95 °C for 15 sec and 60 °C for 1 min, with a final dissociation stage. Relative expression was calculated using the 2^−ΔΔCt^ method [39], and results were presented as fold changes (mean ± standard deviation) in gene expression.

### 2.7. Intracytoplasmic Cytokine Profile Assessed by Flow Cytometry

Flow cytometry was used to analyze intracellular cytokines (IFN-γ, TNF-α, IL-10), following protocols adapted from Machado et al. [38]. Splenocytes (5 × 10^6^ cells per well) were cultured in 96-well round-bottom plates with complete RPMI 1640 medium, where cells were kept unstimulated (medium) or stimulated with SLA (20 µg/well) for 48 h at 37 °C and 5% (*v*/*v*) CO_2_. Brefeldin A (10 µg/well; Sigma-Aldrich, Burlington, MA, USA) was added 4 h after culture initiation to inhibit cytokine secretion. Wells containing PMA (5 ng/well) and ionomycin (1 µg/well) served as positive controls. Cells were stained with Fixable Viability Stain 450 (FVS450, BD Biosciences, Franklin Lakes, NJ, USA) for 15 min at RT, followed by surface staining with antibodies against CD3 (BV650, clone 145-2C11), CD4 (BV605, clone RM4-5), and CD8 (BV786, clone 53–6.7). After fixation and permeabilization with PBS containing 0.5% (*w*/*v*) saponin, cells were incubated with antibodies against IFN-γ (AF700, clone XMG1.2), TNF-α (PE-Cy7, clone LG.3A10), and IL-10 (APC, clone JES5-16E) for 30 min at RT. Samples (100,000 events per sample) were acquired on a BD LSRFortessa™ flow cytometer using FACSDiva™ software.v. 6.1.3 FlowJo™ v11.1 software was used for data analysis. Dead cells were excluded via FVS450 staining, and live cells were gated on CD3^+^CD4^+^ and CD3^+^CD8^+^ populations for intracellular cytokine analysis. Data were expressed as index values, calculated as the ratio of cytokine-positive cells in SLA-stimulated cultures (SC) to those in unstimulated control cultures (CCs). Additionally, IFN-γ/IL-10 ratios were calculated using values from SLA-stimulated cultures.

### 2.8. Humoral Response

The humoral response was evaluated by quantifying the serum levels of anti-ChimT and anti-SLA IgG1 and IgG2a antibodies as previously described [35]. Titration curves were first established to determine the optimal concentrations of antigens and antibody dilutions. Microtiter plates (Jetbiofil^®^, Belo Horizonte, Minas Gerais, Brazil) were coated overnight at 4 °C with ChimT (0.25 µg/well) and SLA (1.0 µg/well) in carbonate–bicarbonate buffer (pH 9.6). Serum samples were diluted 1:100 in PBS-T and added to each well and incubated for 1 h at 37 °C. After washing, horseradish peroxidase (HRP)-conjugated secondary antibodies specific for IgG1 (rat anti-mouse, cat. SA1-35640) or IgG2a (rat anti-mouse, cat. SA1-35646; Invitrogen, Waltham, MA, USA) were diluted 1:10,000 in PBS-T and added to the wells. Colorimetric detection was developed by incubation with 50 μL per well of TMB (3,3′,5,5′ tetramethyl benzidine, Scienco, Lages, Santa Catarina, Brazil) for 7 min in the dark. The reaction was stopped with 2 N H_2_SO_2_, and absorbance was measured at 492 nm using a microplate reader (SpectraMax Plus, Molecular Devices, San Jose, CA, USA).

### 2.9. Renal and Hepatic Toxicity

Organic toxicity was assessed through the quantification of urea and creatinine as indicators of renal function, while alanine transaminase (ALT) and aspartate transaminase (AST) levels were measured as markers of hepatic injury [37]. Serum samples from uninfected and untreated (naive) mice were used as negative controls. The measurements were performed using commercial colorimetric kits (Labtest Diagnóstica^®^, Lagoa Santa, Minas Gerais, Brazil), according to the manufacturer’s instructions.

### 2.10. Parasite Load by Limiting Dilution Technique

Parasite burden in the liver, spleen, bone marrow (BM), and draining lymph nodes (dLNs) was quantified by limiting dilution, as previously described [35]. Briefly, tissues were weighed and homogenized in sterile PBS using a glass tissue grinder (Wertheim, Germany). Homogenates were centrifuged at 150× *g* to remove debris, and the supernatants were further centrifuged at 2000× *g* to pellet the cells. Pellets were suspended in complete Schneider’s medium, and 220 µL of each sample was distributed into 96-well flat-bottom microplates (Nunc) in triplicate. Serial 10-fold dilutions (from 10^−1^ to 10^−12^) were prepared in complete Schneider’s medium, and cultures were incubated at 24 °C. After 7 days, the highest dilution with viable promastigotes was recorded, and results were expressed as the negative log of the last positive dilution per milligram of tissue.

### 2.11. Parasite Load Estimated by Quantitative PCR (qPCR)

The splenic parasite load was also evaluated by qPCR as previously described [40]. After the animals were euthanized, the total genomic DNA was extracted from approximately 15 to 30 mg of splenic tissue using the Wizard^®^ Genomic DNA Purification Kit (Promega, Madison, WI, USA) according to the manufacturer’s instructions and suspended in UltraPure™ DNase/RNase-Free Distilled Water (Invitrogen, Waltham, MA, USA). The concentration of DNA obtained was determined with a NanoDrop spectrophotometer 2000 (Thermo Scientific, Waltham, MA, USA), and the quality of the samples was measured using a 260/280 nm ratio between 1.8 and 2.0. A standard curve was produced using *L. infantum* DNA (MHOM/BR/1974/PP75 strain) extracted from 1 × 10^8^ promastigotes by using the CTAB (cetyltrimethylammonium bromide) method. After elution, the DNA pellet was extracted in 100 µL of autoclaved ultrapure water, and the concentration was adjusted for 10^6^ parasites. From there, serial dilutions were made from 10×, obtaining seven points on the curve. The following primers were used: *L. infantum* DNA: forward: 5′-CCTATTTTACACCAACCCCCAGT-3′; reverse: 5′-GGGTAGGGGCGTTCTGCGAAA-3′. Mouse β-actin (internal control): forward: 5′-CAGAGCAAGAGAGGTATCC-3′; reverse: 5′-TCATTGTAGAAGGTGTGGTGC-3′. The reactions contained 5 µL of Master Mix, 2 mM of each primer, and 4 µL of DNA (25 ng/µL). Cycling conditions were an initial denaturation at 95 °C for 10 min, followed by 40 cycles at 95 °C for 15 sec and 60 °C for 1 min. Reactions were developed in 96-well plates using the 7500 HT Real-Time PCR System (Applied Biosystems, Waltham, MA, USA) with Power Up SYBR™ Green PCR Master Mix (Thermo Fisher, Waltham, MA, USA). Each 96-well reaction plate contained the standard curve in triplicate (efficiency, 97.5%; r^2^ 0.99) in duplicate samples, a negative control (no DNA) and control genes. Results were expressed quantitatively as the number of amastigotes per milligram of splenic tissue multiplied by the total weight of the organ.

### 2.12. Statistical Analysis

Data were compiled using Microsoft Excel (version 10.0) and analyzed using GraphPad Prism version 10.0.2 for Windows (www.graphpad.com; GraphPad Software Inc., La Jolla, CA, USA). An ordinary one-way analysis of variance (ANOVA) followed by Tukey’s multiple comparison test, which assumes a normal Gaussian distribution and a single pooled variance, was used for comparisons between the groups. Differences were considered statistically significant with *p* < 0.05. The vaccination experiments were repeated twice and the results were similar between them. In each experiment, eight animals per group were used, indicating a total of sixteen animals per experimental group. Data shown in this study are representative of the first experiment. Results obtained in the second study are shown at https://doi.org/10.6084/m9.figshare.30380200.v1 (accessed on 14 November 2025).

## 3. Results

### 3.1. Cytokine and Antibody Responses Following Treatment

**Figure 1:**
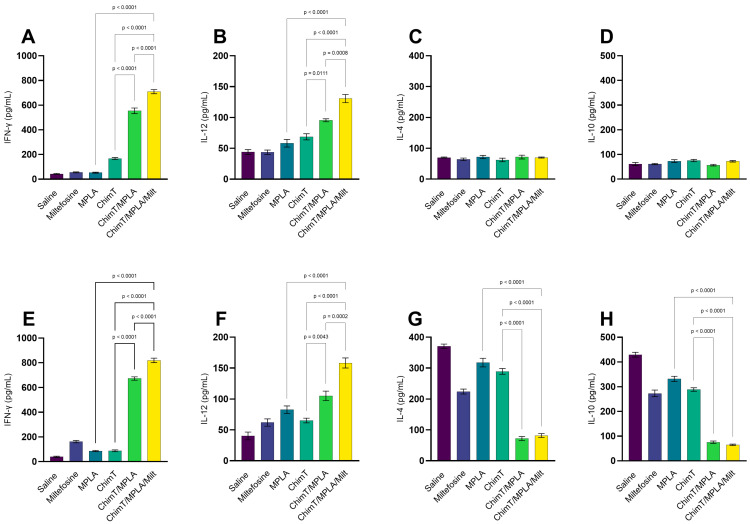
Cytokine production in animals obtained one day after treatment. Mice were infected and later received saline, miltefosine (Milt), ChimT, MPLA, ChimT/MPLA, or ChimT/MPLA/Milt. One day after treatment, their spleen cells (5 × 10^6^ per well) were cultured and kept unstimulated (control) or stimulated with ChimT (10 µg/well) or SLA (25 µg/well) for 48 h at 37 °C in 5% CO_2_. Culture supernatants were collected and used to quantify IFN-γ, IL-4, IL-10, and IL-12 cytokines through capture ELISA using commercial kits. Results obtained 1 (panels (**A**–**D**)) and 30 (panels (**E**–**H**)) days after treatment are shown. Bars indicate the mean ± standard deviation of the groups. Statistical differences between the groups are indicated with the respective *p*-value.

**Figure 2:**
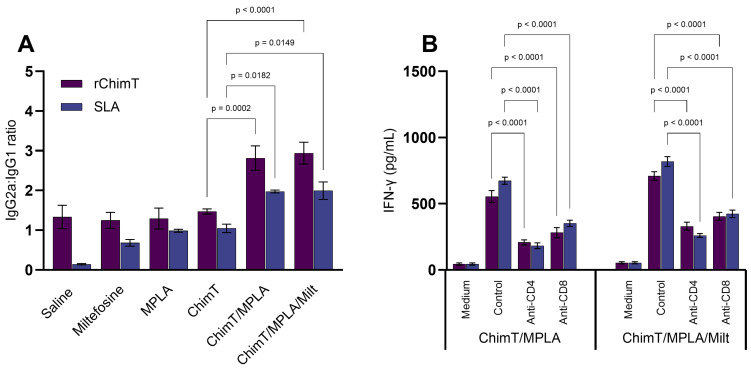
Antibody production and IFN-γ secretion in cell cultures one day after therapy. Mice were infected and later received saline, miltefosine (Milt), ChimT, MPLA, ChimT/MPLA, or ChimT/MPLA/Milt. One day after treatment, sera samples of animals were collected and used to evaluate levels of anti-ChimT and anti-SLA IgG1 and IgG2a antibodies. With the optical density values, ratios between IgG2a and IgG1 data were calculated and results are shown (panel (**A**)). Also, spleen cells from ChimT/MPLA and ChimT/MPLA/Milt groups were stimulated with SLA and treated with either mouse anti-CD4 or anti-CD8 antibody for 48 h at 37 °C in 5% CO_2_. IFN-γ production was evaluated and supernatant was collected and used to quantify using the commercial kit (panel (**B**)). Bars indicate the mean ± standard deviation of the groups. Statistical differences between the groups are indicated with the respective *p*-value.

The immune response profile was assessed 1 and 30 days after treatments. After one day, mice receiving ChimT/MPLA and ChimT/MPLA/Milt exhibited more polarized Th1-type cytokine production compared with the other groups. This was characterized by more elevated levels of IFN-γ and IL-12 in cell culture supernatants, after stimulation with ChimT (Figure 1A–D) and SLA (Figure 1E–H). By contrast, the saline group mice exhibited higher levels of anti-parasite IL-4 and IL-10 cytokines, indicative of a Th2-type response. Serum antibody levels were also evaluated and mice treated with ChimT/MPLA and ChimT/MPLA/Milt produced higher levels of IgG2a isotype antibodies specific to ChimT and SLA, compared with IgG1 levels, which are reflected in higher IgG2a/IgG1 ratios, indicative of a Th1-skewed humoral response (Figure 2A). To indirectly confirm the T cell subtypes that were involved in IFN-γ production, spleen cell cultures were incubated with either anti-CD4 or anti-CD8 monoclonal antibodies, and subsequent cytokine production was measured. The results showed that both antibody depletions significantly reduced IFN-γ secretion when compared with untreated cell cultures, confirming the contribution of both T cell subtypes (Figure 2B).

**Figure 3:**
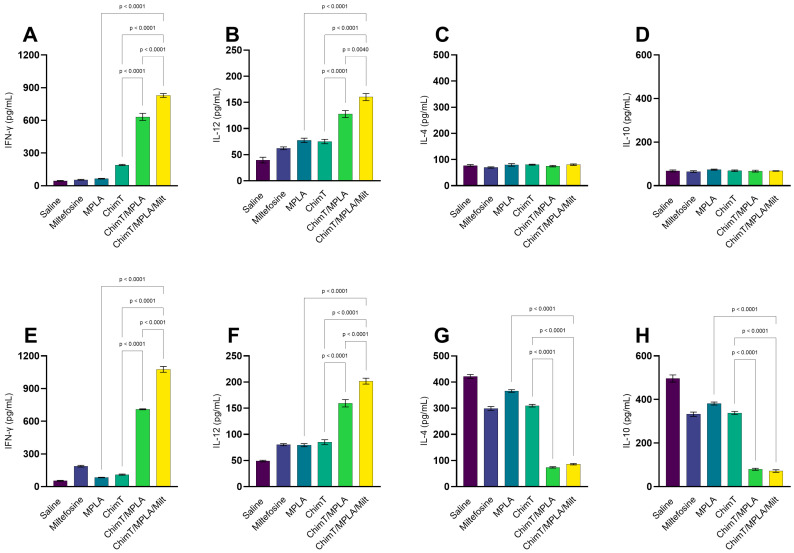
Cytokine production generated 30 days after treatment. Mice were infected and later received saline, miltefosine (Milt), ChimT, MPLA, ChimT/MPLA, or ChimT/MPLA/Milt. Thirty days after treatment, their spleen cells (5 × 10^6^ per well) were cultured and kept unstimulated (control) or stimulated with ChimT (10 µg/well) or SLA (25 µg/well) for 48 h at 37 °C in 5% CO_2_. Culture supernatants were collected and used to quantify IFN-γ, IL-4, IL-10, and IL-12 cytokines through capture ELISA using commercial kits. Results obtained 1 (panels (**A**–**D**)) and 30 (panels (**E**–**H**)) days after treatment are shown. Bars indicate the mean ± standard deviation of the groups. Statistical differences between the groups are indicated with the respective *p*-value.

**Figure 4:**
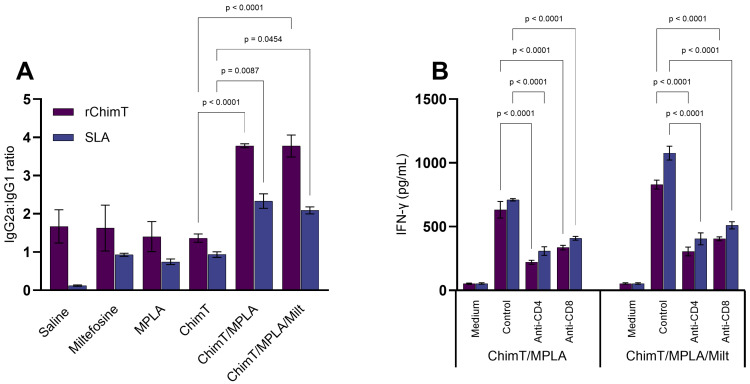
Humoral response and IFN-γ production in treated cell cultures 30 days after therapy. Mice were infected and later received saline, miltefosine (Milt), ChimT, MPLA, ChimT/MPLA, or ChimT/MPLA/Milt. Thirty days after treatment, sera samples of animals were collected and used to evaluate levels of anti-ChimT and anti-SLA IgG1 and IgG2a antibodies. With the optical density values, ratios between IgG2a and IgG1 data were calculated and results are shown (panel (**A**)). Also, spleen cells from ChimT/MPLA and ChimT/MPLA/Milt groups were stimulated with SLA and treated with either mouse anti-CD4 or anti-CD8 antibodies for 48 h at 37 °C in 5% CO_2_. IFN-γ production was evaluated and supernatant was collected and used to quantify using the commercial kit (panel (**B**)). Bars indicate the mean ± standard deviation of the groups. Statistical differences between the groups are indicated with the respective *p*-value.

The immune profile persisted at 30 days post treatment, with the ChimT/MPLA and ChimT/MPLA/Milt groups maintaining higher levels of IFN-γ and IL-12, whilst the saline group mice displayed predominantly IL-4 and IL-10 cytokine production, both specific to ChimT (Figure 3A–D) and SLA (Figure 3E–H). The Th1-associated antibody profile (i.e., elevated IgG2a and IgG2a/IgG1 ratios) was also maintained in the ChimT/MPLA and ChimT/MPLA/Milt groups at this later point (Figure 4A). Additionally, when blocking with either the anti-CD4^+^ or CD8^+^ T cell monoclonal antibody, lower IFN-γ production was maintained in these groups, suggesting the involvement of both T cell subsets in the long-term production of this cytokine (Figure 4B).

### 3.2. IFN-γ mRNA Expression and Nitrite Production

**Figure 5:**
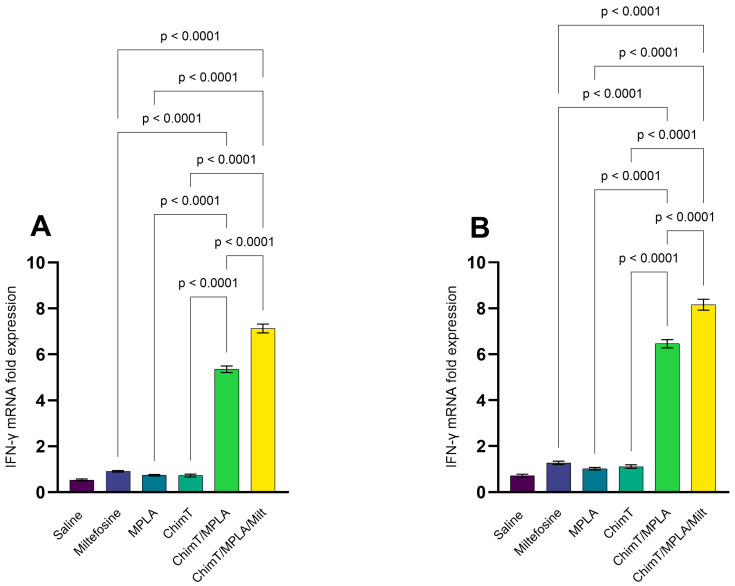
IFN-γ mRNA expression evaluated through RT-qPCR. Mice were infected and later received saline, miltefosine (Milt), ChimT, MPLA, ChimT/MPLA, or ChimT/MPLA/Milt. One and thirty days after treatment, they were euthanized and their spleen cells (5 × 10^6^ per well) were either unstimulated (control) or stimulated with ChimT (10 µg/well) or SLA (25 µg/well) for 48 h at 37 °C in 5% CO_2_. RNA content was extracted and used to evaluate the IFN-γ mRNA expression in stimulated cultures through RT-qPCR. Results obtained 1 (panel (**A**)) and 30 (panel (**B**)) days after treatment are shown. Bars indicate the mean ± standard deviation of the groups. Statistical differences between the groups are indicated with the respective *p*-value.

**Figure 6:**
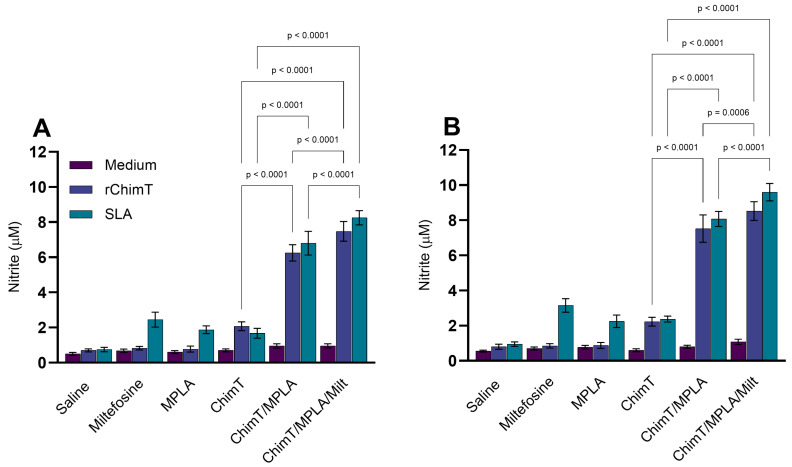
Nitrite secretion evaluated 1 and 30 days after treatment. Mice were infected and later received saline, miltefosine (Milt), ChimT, MPLA, ChimT/MPLA, or ChimT/MPLA/Milt. One and thirty days after treatment, they were euthanized and their spleen cells (5 × 10^6^ per well) were either unstimulated (control) or stimulated with ChimT (10 µg/well) or SLA (25 µg/well) for 48 h at 37 °C in 5% CO_2_. Cell culture supernatants were collected and used to evaluate the nitrite secretion through Griess reaction. Results obtained 1 (panel (**A**)) and 30 (panel (**B**)) days after therapy are shown. Bars indicate the mean ± standard deviation of the groups. Statistical differences between the groups are indicated with the respective *p*-value.

**Figure 7:**
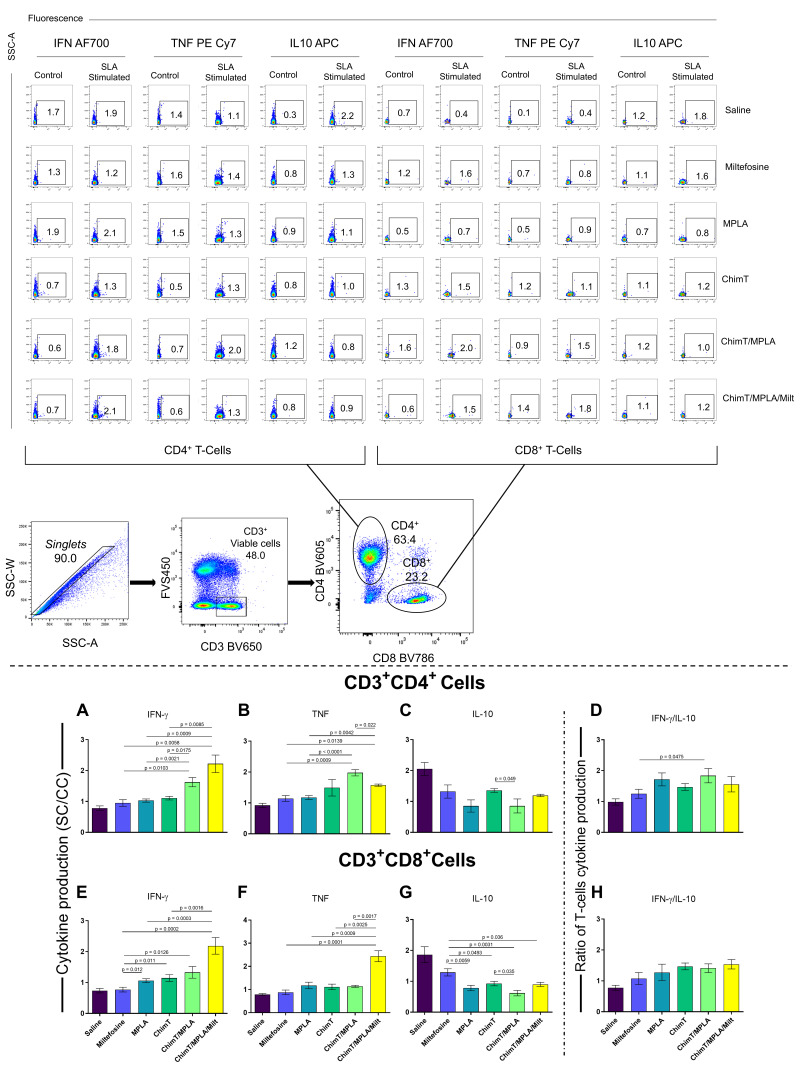
Intracytoplasmic cytokine profile in SLA-stimulated cell cultures evaluated through flow cytometry. Plots of the gating strategy to characterize the frequency (percentage) of T cells producing intracellular cytokines: IFN-γ, TNF-α, and IL-10 in mice groups are shown (**upper** panel). Plots representing the IFN-γ, TNF-α, and IL-10 production specific to CD4^+^ (panels (**A**), (**B**), and (**C**), respectively) and CD8^+^ (panels (**E**), (**F**), and (**G**), respectively) T cells are shown. In addition, IFN-γ/IL-10 ratios were calculated for CD4^+^ and CD8^+^ T cells, respectively (panels (**D**,**H**)). Bars indicate the mean ± standard deviation of the indexes of cytokine production, which were calculated based on SLA-stimulated cultures (SCs) divided by the control culture (CC). Statistical differences between the groups are indicated with the respective *p*-value.

The expression of IFN-γ mRNA in ChimT- and SLA-stimulated cell cultures was up-regulated in mice treated with ChimT/MPLA and ChimT/MPLA/Milt, compared with data from saline group mice, at both 1 (Figure 5A) and 30 (Figure 5B) days post treatment. Nitrite production, a proxy marker of nitric oxide synthesis, was also higher in both groups at both time points following cell stimulation (Figure 6A,B). Flow cytometric analysis confirmed that the ChimT/MPLA and ChimT/MPLA/Milt groups had elevated frequencies of IFN-γ- and TNF-α-producing CD4^+^ and CD8^+^ T cells, while the saline-receiving mice showed a predominance of IL-10-producing T cells (Figure 7). The IFN-γ/IL-10 ratios were also highest in ChimT/MPLA and ChimT/MPLA/Milt groups, further supporting the presence of a Th1-biased immune response.

### 3.3. Toxicological Assays in Treated Mice

**Figure 8:**
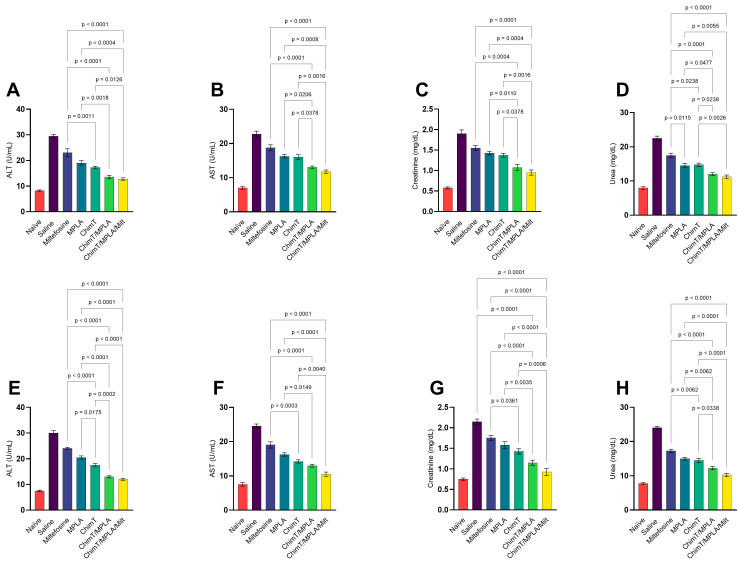
In vivo toxicity evaluated 1 and 30 days after treatment. Mice were infected and later received saline, miltefosine (Milt), ChimT, MPLA, ChimT/MPLA, or ChimT/MPLA/Milt. One and thirty days after treatment, they were euthanized and their sera samples were collected to evaluate the levels of urea, creatinine, alanine aminotransferase (ALT), and aspartate aminotransferase (AST). Sera were also collected from uninfected and untreated (naive) mice, which were used as negative controls. Results obtained 1 (panels (**A**–**D**)) and 30 (panels (**E**–**H**)) days after treatment are shown. Bars indicate the mean ± standard deviation of the groups. Statistical differences between the groups are indicated with the respective *p*-value.

The levels of urea and creatinine (renal function markers) and ALT and AST (hepatic function markers) were measured in mouse serum samples to evaluate organic toxicity. At both 1 (Figure 8A–D) and 30 (Figure 8E–H) days post treatment, mice receiving ChimT/MPLA and ChimT/MPLA/Milt exhibited significantly lower levels of these biomarkers compared, in particular, with the control saline group, indicating minimal renal and hepatic toxicity. Notably, the administration of ChimT/MPLA/Milt to uninfected mice was non-toxic. ChimT, ChimT/MPLA, and Milt also lowered the levels of urea, creatinine, ALT, and AST enzymes compared with the saline group, although these biomarker levels were still higher than those measured for the ChimT/MPLA and ChimT/MPLA/Milt groups.

### 3.4. Parasite Burden Measured in Treated Mice

**Figure 9:**
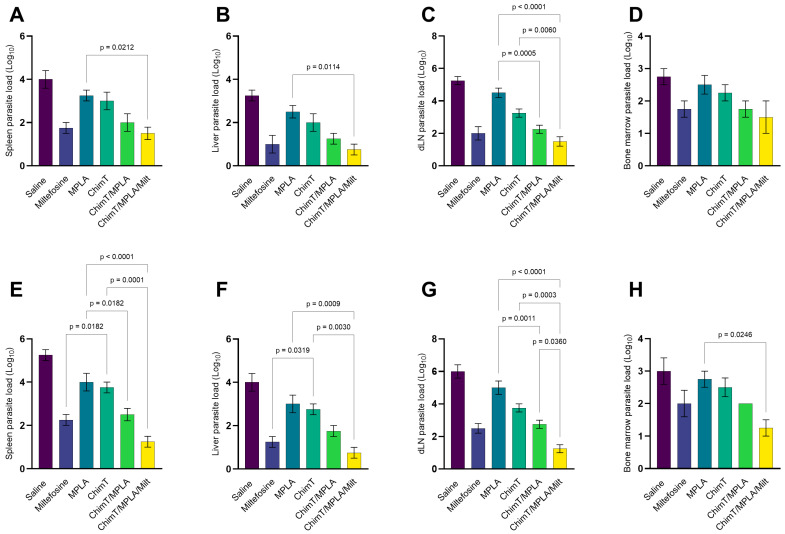
Parasite load estimated by limiting dilution technique. Mice were infected and later received saline, miltefosine (Milt), ChimT, MPLA, ChimT/MPLA, or ChimT/MPLA/Milt. One and thirty days after treatment, they were euthanized and their livers, spleens, draining lymph nodes (dLNs), and bone marrows (BM) were collected and used in a limiting dilution technique to estimate the parasite burden. Results obtained 1 (panels (**A**–**D**)) and 30 (panels (**E**–**H**)) days after treatment are shown. Bars indicate the mean ± standard deviation of the groups. Statistical differences between the groups are indicated with the respective *p*-value.

The parasite load in the liver, spleen, BM, and dLNs was evaluated through the limiting dilution assay at 1 (Figure 9A–D) and 30 (Figure 9E–H) days post treatment. At both time points, mice treated with ChimT/MPLA and ChimT/MPLA/Milt exhibited significantly reduced parasite loads across the evaluated organs compared with saline group mice. Using miltefosine alone also reduced internal parasitism, thus demonstrating the known antileishmanial efficacy of this drug. The association between ChimT, MPLA, and Milt appeared to have an additive effect compared with the use of two or three products, with a lower parasite burden found in the organs of treated mice.

We also completed the quantification of the parasite burden in the animals’ spleens through qPCR assay, and the results obtained with experiments performed one day after treatment showed that ChimT/MPLA/Milt group mice presented a mean value of 0.9-log amastigotes per mg of splenic tissue, while mice receiving saline, MPLA, ChimT, Milt, or ChimT/MPLA presented values on the order of 2.9-, 2.4-, 1.9-, 1.3-, and 1.2-log amastigotes per mg of tissue, respectively. Thirty days after treatment, the value of 1.1-log amastigotes per mg of splenic tissue was found in the ChimT/MPLA/Milt group, while animals receiving saline, MPLA, ChimT, Milt, and ChimT/MPLA presented values of 3.6-, 2.9-, 2.4-, 1.4-, and 1.3-log amastigotes per mg of tissue, respectively.

## 4. Discussion

There is no vaccine for human VL, and drug treatment is toxic, expensive, and associated with the emergence of resistant strains [4,41]. Thus, alternative control measures for VL are necessary and immunotherapy, which combines the use of antileishmanial drugs with immunogenic vaccines, is a promising strategy [42,43]. The principle behind immunotherapy is that drugs reduce the parasite load in infected hosts, whilst the immunogens stimulate a protective T cell-dependent immune response [44,45]. In the current study, we combined ChimT, a recombinant chimeric protein that is prophylactic [35] and immunotherapeutic [36] against VL, with MPLA, as an adjuvant, and miltefosine, as the antileishmanial drug. Our goal was to determine if this new combination could reduce parasitism in *L. infantum*-infected mice. The key findings from our study were that ChimT/MPLA/Milt (i) was highly effective in reducing the parasite load within mouse internal organs; (ii) induced Th1-type cellular and humoral immune responses; (iii) was non-toxic and concomitantly improved the health status of infected animals.

Several recombinant antigens have been exploited as vaccine candidates against experimental VL, and they have been selected due to their importance for parasite survival or pathogenicity. However, only a few antigens are available to protect dogs and there is no licensed human vaccine. Equally important is the incorporation of an appropriate adjuvant for increasing the strength and duration of the immune response against parasite antigens, ensuring proper cellular activation and promoting a Th1-type cell-mediated immune response, all of which are essential attributes against intracellular pathogens, including *Leishmania* [46,47]. In the present study, we used ChimT associated with MPLA as an adjuvant. The protein used alone provided lower protective immunity, but when associated with MPLA, the immunological and parasitological profile yielded a better prognosis for the infected animals. Other studies have also shown the requirement of adjuvants in compositions involving recombinant proteins with prophylactic [48,49] or therapeutic [25,50] purposes against VL, demonstrating the importance of this association to induce effective host immunity. ChimT was recently used in immunotherapy in combination with MPLA and AmpB against murine VL [36]. Although this combination was effective, the toxicity of AmpB was a significant contra-indication. In the current study, miltefosine associated with ChimT/MPLA did not induce organic toxicity in the treated mice and highlights the potential of this immunotherapy for in future studies in other mammalian models.

The development of prophylactic or therapeutic vaccination is required as a control measure against infectious diseases. Adjuvants are necessary to increase both the antigenicity and immunogenicity of candidate vaccine antigens. MPLA is the detoxified derivative of lipopolysaccharide from *Salmonella minnesota*, which stimulates the activation of innate immunity via Toll-*like* receptor 4 (TLR-4), directly activating antigen-presenting cells (APCs) expressing TLR-4, which in turn stimulates the production of pro-inflammatory cytokines and co-stimulatory molecules [50,51]. MPLA favors the induction of APCs producing IFN-γ and switching antibody isotype towards IgG2a antibodies. MPLA has been evaluated in several vaccine candidates against leishmaniasis, since Th1-type humoral and cellular responses are required for protection against infection [52,53]. In our study, the ChimT protein given alone to infected animals was poorly immunogenic and, consequently, a weak th1-type response was developed. However, the addition of MPLA stimulated a more potent pro-inflammatory response, characterized by the production of high levels of IFN-γ, IL-12, TNF-α, nitrite, and IgG2a isotype antibodies. The up-regulation of these biological responses contributed to controlling parasitism in treated and infected animals. Thus, these data support the hypothesis that immune adjuvants must be included within vaccines and highlights the use of MPLA plus ChimT as an effective immunogenic combination for mammals.

*Leishmania* infection causes a wide spectrum of disease outcomes, ranging from asymptomatic to fatal infections, which are dependent on parasite infectivity and differences in host susceptibility [54]. An early immune response involving the development of Th1-type CD4^+^ and CD8^+^ T cells can resolve infection and produce long-lasting immunity. IFN-γ expression is associated with resolving infection in mammals, since this cytokine induces parasite killing by macrophages following nitric oxide release, as well as causing the direct inhibition of IL-4 and IL-10 production [55,56,57,58,59,60,61]. Despite the progress in *Leishmania* vaccine development, complete immune protection is not facile due to the lack of persistent parasites and sterile immunity, since parasite antigens are not effective in inducing long-term immune memory. Another challenge to achieving long-lasting protection is the lack of immunodominant antigens that can be recognized by T cells over long periods of time. Studies have shown that immune memory generated after secondary infection with *Leishmania* is important for conferring and maintaining protection through central memory T cells [62]. Indeed, these cells become tissue-homing effector cells, which induce protection against re-infection [63]. In the present study, we did not evaluate the development of memory T cells in treated animals. However, because mice receiving ChimT/MPLA/Milt showed clear parasitological regression and a strong Th1-type response 30 days after treatment, we can hypothesize that a long-lasting protective immunity is developing that could induce an appropriate secondary response and protect against re-infection. In this context, although additional experiments are required to evaluate whether ChimT/MPLA/Milt therapy can induce a strong and sustained Th1-type response in treated rodents, we can speculate over the potential long duration and memory responses related to the administration of ChimT/MPLA/Milt. This has potential importance for translation to combat leishmaniasis in other hosts, such as humans and dogs.

In this study we replaced AmpB as the antileishmanial drug with miltefosine. Immunotherapeutics studies combining immunogens and miltefosine have been developed against VL: for example, Carvalho et al. [43] associated the drug with the LBSap vaccine and saponin as an adjuvant to treat *L. infantum*-infected hamsters. Treated hamsters showed an improvement in their health and the development of an effective Th1-type immune response to combat infection. Santana et al. [64] also combined miltefosine with the recombinant *Leishmania* cysteine proteinase to treat *L. infantum*-infected hamsters. The combination reduced organ parasitism in treated animals, accompanied by the development of a protective Th1-type response. Similar findings were reported by Gupta et al. [65], who combined class-B bacterial CpG-ODN and miltefosine to treat *L. donovani*-infected animals. Comparatively, our data shows that the effective antileishmanial action of miltefosine is supported by the literature and highlights the potential anti-*Leishmania* role of this drug in mammals, which results in direct parasite elimination independent of an immune response [10,66,67].

Our results showed that miltefosine-treated mice also presented low levels of organic parasitism, when both limiting dilution and qPCR assays were performed one day after therapy. Such data were similar to those found in ChimT/MPLA/Milt-receiving mice. When evaluations were performed after a longer period of time after the treatment, a slightly higher parasitism was found in miltefosine-treated animals compared with the ChimT/MPLA/Milt group. These results could indicate a possible increase in organic parasitism in the miltefosine group, compared with values found in animals receiving the combination, mainly due to the absence of an immunogen to reactivate the susceptible immune response present in the treated animals. In fact, it has been shown that the success of a long-term antileishmanial treatment depends on the combined action of an effective drug followed by the activation of the host’s immune response [42]. Although we have not performed parasitological assays longer than 30 days after therapy, one could speculate that the increase in the organic parasitism from miltefosine-treated mice could occur after this period of time. This fact can be corroborated by the lowest IFN-γ and highest IL-4 and IL-10 levels found in miltefosine-treated mice compared with cytokine data obtained in the ChimT/MPLA/Milt group. In this context, the association between the antileishmanial drug to eliminate parasites in a direct way and an immunogen to revert the susceptibility immune profile in treated animals can work together and contribute to a longer and sustained therapeutic response against re-infection by the parasites still present in the host’ body [22].

In VL, there is a significant loss of cellular and tissue integrity in internal organs due to a high parasite burden; this contributes to a deteriorating clinical picture leading to a series of aggravations and culminating in multiple organ failure [68,69]. Glomerular and tubular functions during active VL are altered, and infected hosts can develop proteinuria, haematuria, abnormalities in urinary concentration, acidification, and acute and chronic renal insufficiency. Moreover, drug therapy can contribute to toxicity, e.g., AmpB can cause renal, cardiac, and hepatic toxicity [70]. We analyzed renal and hepatic damage markers in this study and found that the administration of ChimT/MPLA/Milt was associated with significant reductions in the levels of urea, creatinine, ALT, and AST, compared with the levels in control mice. We deduced that the increasing parasite burden found in the saline group mice contributed to the increase in organ damage, which resulted in a higher production of enzymes and disease progression. Conversely, miltefosine reduced parasitism, but without causing organ toxicity and, consequently, showed possibly better biological activity than AmpB. Although additional studies are required to confirm that miltefosine is non-toxic, the promising data generated herein should stimulate further studies, such as using the drug plus an immunogen to treat VL.

There are some limitations of this study that need to be reported; these include the (i) absence of dose–response curves, with the aim of reducing the number of doses and/or their concentrations; (ii) need for additional immunological, parasitological, and biochemical assays to prove the effective therapeutic response induced by ChimT/MPLA/Milt; (iii) need for comprehensive histopathological analyses of the key affected organs of the animals, such as the liver, kidney, spleen, etc. Addressing these limitations would complement the data from this current study and directly demonstrate tissue integrity and the absence of inflammation following treatment with ChimT/MPLA/Milt.

## 5. Conclusions

The data from this study highlight the possibility of using ChimT/MPLA/Milt as a potential immunotherapy for VL. This combination induced a polarized Th1-type cellular and humoral immune response in treated animals, which contributed to significant reductions in organ parasitism, accompanied by lower toxicity caused by chronic *Leishmania* infection. The treatment produced consistent results for 1 and 30 days after therapy and demonstrates that ChimT/MPLA/Milt deserves further development as an immunotherapy against *L. infantum* infection in mammals.

## Data Availability

The data obtained from the second experiment with treated and infected mice regarding the in vivo toxicity assay, the cellular and humoral immune responses, the IFN-γ mRNA expression, and the parasite burden estimated through the limiting dilution technique and by qPCR, 1 and 30 days after treatment can be found at https://doi.org/10.6084/m9.figshare.30380200.v1 (accessed 10 November 2025).

## Supplementary Materials

The following supporting information can be downloaded at: https://www.mdpi.com/article/10.3390/pathogens14121202/s1, Supplementary Figure S1: Production of the recombinant protein.; Supplementary Figure S2: Experimental groups and treatment protocols used in the study.
