# Supplementary figures and images for: Immunotherapeutics Combining a Recombinant Chimeric Protein, Monophosphoryl Lipid A, and Miltefosine Against Visceral Leishmaniasis
Source: Pathogens. 2025 Nov 25;14(12):1202. doi: 10.3390/pathogens14121202 (PMC12736017; doi:10.3390/pathogens14121202)

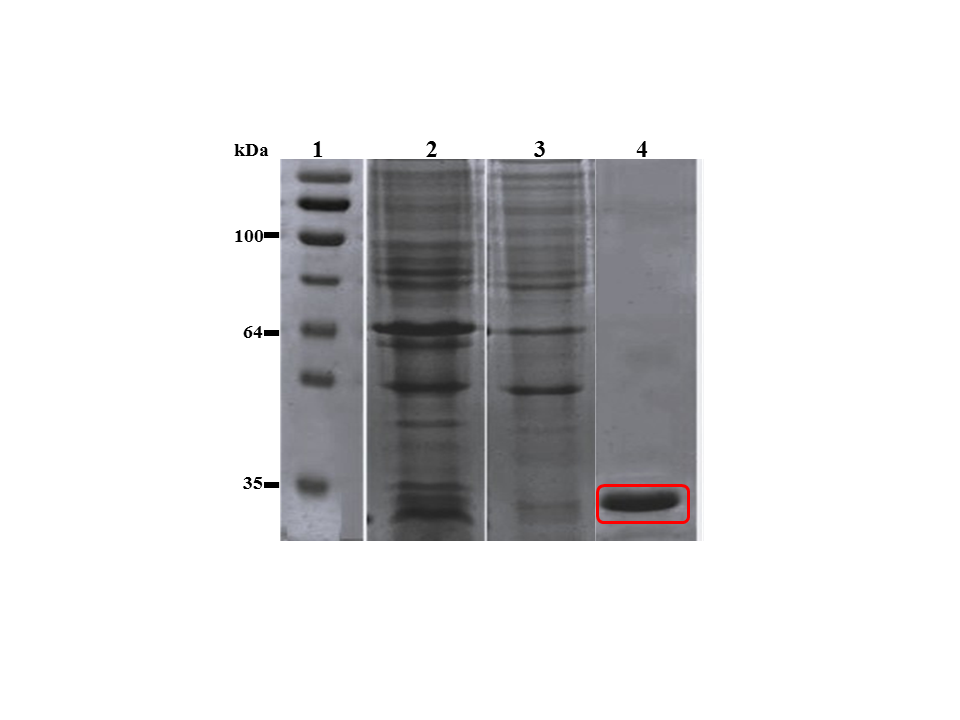

Supplement: Supplementary file 1 [file pathogens-14-01202-s001.zip › Supplementary figure 1.tif]

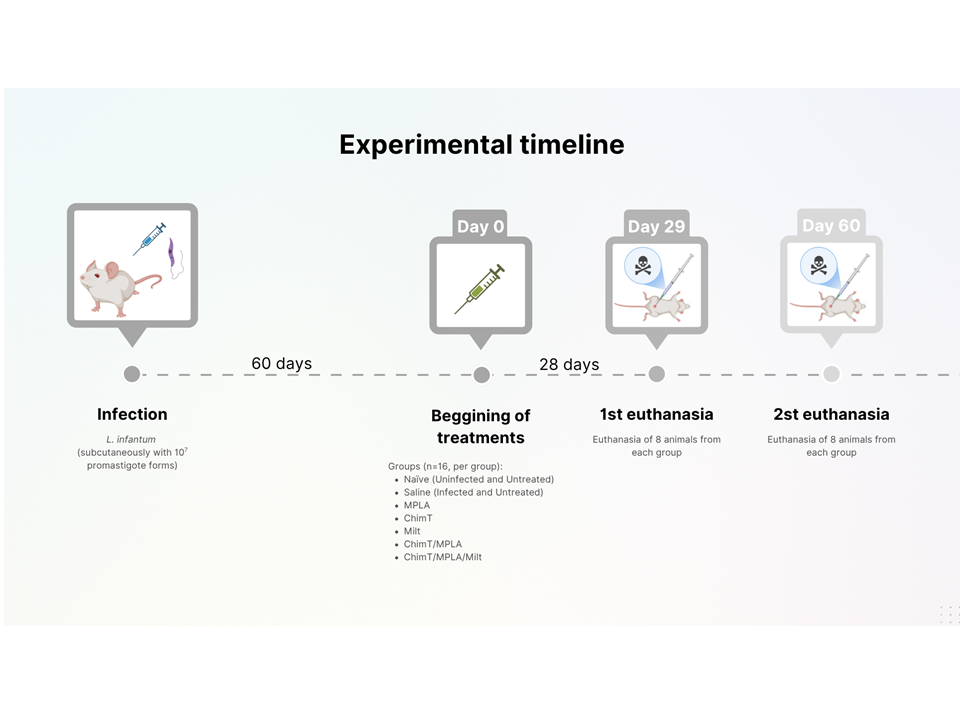

Supplement: Supplementary file 1 [file pathogens-14-01202-s001.zip › Supplementary figure 2.tif]
